# A ^29^Si, ^1^H, and ^13^C Solid-State NMR Study on the Surface Species of Various Depolymerized Organosiloxanes at Silica Surface

**DOI:** 10.1186/s11671-019-2982-2

**Published:** 2019-05-14

**Authors:** Iryna S. Protsak, Yevhenii M. Morozov, Wen Dong, Zichun Le, Dong Zhang, Ian M. Henderson

**Affiliations:** 1 College of Environment, Zhejiang University of Technology, Hangzhou, 310014 China; 2 College of Science, Zhejiang University of Technology, Hangzhou, 310023 China; 3 Key Laboratory of Microbial Technology for Industrial Pollution Control of Zhejiang Province, Hangzhou, 310014 China; 4 Institute for Information Recording of NAS of Ukraine, Kiev, 03113 Ukraine; 5 College of Materials Science & Engineering, Zhejiang University of Technology, Hangzhou, 310014 China; 6 Omphalos Bioscience, LLC, Albuquerque, New Mexico 87110 USA

**Keywords:** Silicones, Dialkyl carbonates, ^1^H solid-state NMR spectroscopy, ^29^Si solid-state NMR spectroscopy, ^13^C solid-state NMR spectroscopy, Surface modification, Fumed nanosilica, Bonding density

## Abstract

**Abstract:**

Three poly(organosiloxanes) (hydromethyl-, dimethyl-, and epoxymethylsiloxane) of different chain lengths and pendant groups and their mixtures of dimethyl (DMC) or diethyl carbonates (DEC) were applied in the modification of fumed silica nanoparticles (FSNs). The resulting modified silicas were studied in depth using ^29^Si, ^1^H, and ^13^C solid-state NMR spectroscopy, elemental analysis, and nitrogen adsorption-desorption (BET) analysis. The obtained results reveal that the type of grafting, grafting density, and structure of the grafted species at the silica surface depend strongly on the length of organosiloxane polymer and on the nature of the “green” additive, DMC or DEC. The spectral changes observed by solid-state NMR spectroscopy suggest that the major products of the reaction of various organosiloxanes and their DMC or DEC mixtures with the surface are D (RR’Si(O_0.5_)_2_) and T (RSi(O_0.5_)_3_) organosiloxane units. It was found that shorter methylhydro (PMHS) and dimethylsiloxane (PDMS) and their mixtures with DMC or DEC form a denser coverage at the silica surface since *S*_BET_ diminution is larger and grafting density is higher than the longest epoxymethylsiloxane (CPDMS) used for FSNs modification. Additionally, for FSNs modified with short organosiloxane PMHS/DEC and also medium organosiloxane PDMS/DMC, the dense coverage formation is accompanied by a greater reduction of isolated silanols, as shown by solid-state ^29^Si NMR spectroscopy, in contrast to reactions with neat organosiloxanes. The surface coverage at FSNs with the longest siloxane (CPDMS) greatly improves with the addition of DMC or DEC. The data on grafting density suggest that molecules in the attached layers of FSNs modified with short PMHS and its mixture of DMC or DEC and medium PDMS and its mixture of DMC form a “vertical” orientation of the grafted methylhydrosiloxane and dimethylsiloxane chains, in contrast to the reaction with PDMS/DEC and epoxide methylsiloxane in the presence of DMC or DEC, which indicates a “horizontal” chain orientation of the grafted methyl and epoxysiloxane molecules. This study highlights the major role of solid-state NMR spectroscopy for comprehensive characterization of solid surfaces.

**Graphical abstract:**

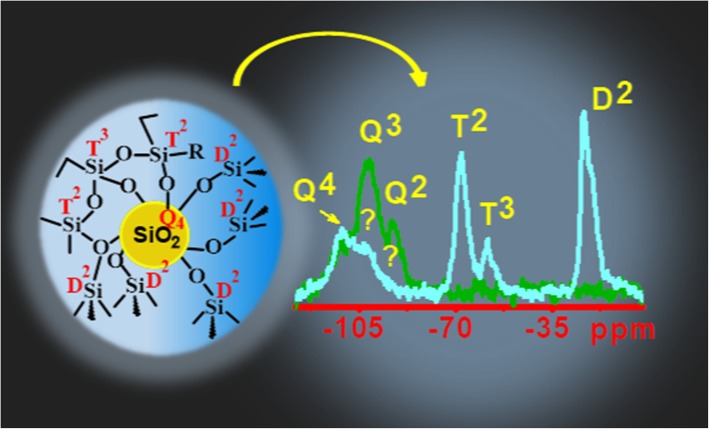

**Electronic supplementary material:**

The online version of this article (10.1186/s11671-019-2982-2) contains supplementary material, which is available to authorized users.

## Introduction

Hydrophobized fumed silica nanoparticles (FSNs) are of interest from a practical point of view because these materials can be better fillers of nonpolar or weakly polar polymers or more appropriate hydrophobic materials for other practical applications than unmodified hydrophilic silica [1–4]. Functionalization of FSNs can be performed using various traditional types of modifying agents such as alkoxy-, halo-, and aminosilanes and organosilazanes [3–8]. However, due to the high reactivity and moisture sensitivity of the modifying agents, purification is often critical for these hydrolyzable precursors. Organosiloxanes with methyl-terminated groups provide a viable and environmentally benign alternative to the chemical functionalization of oxides, taking into account three aspects of their structure that set it apart from carbon-based polymers: the bond lengths of Si–O and Si–C (1.63 and 1.90 Å) in organosiloxane are longer than the C–C (1.53 Å) bonds of most polymers; the S–O–Si bond angle (143°) is significantly greater than the C–C–C bond angles (109°) in the main chain of carbon-based polymers; and the differences in Pauling electronegativity values between silicon (1.8) and oxygen (3.5) and between silicon (1.8) and carbon (2.5) impart ionic character to both the Si–O backbone bonds (51% ionic) and the Si–C bonds (12% ionic). These three structural differences allow rotational and vibrational degrees of freedom in organosiloxane that are not available to carbon-based polymers and are the basis for unusual and unique properties: high thermal stability; excellent dielectric properties; and resistance to oxygen, water, and UV irradiation and so on [5, 8–11]. Linear organosiloxanes are generally not considered to be reactive with inorganic oxide surfaces, and an enormous research effort has been made over the last 50 years to develop other silicon-containing reagents with reactive functional groups [12]. One of the likely ways to increase the reactivity of a silicone polymer is partial depolymerization of high molecular poly(organosiloxanes), followed by grafting formed oligomers (with terminated alkoxy groups) on silica surfaces. Complete depolymerization of poly(dimethylsiloxanes) can be achieved by treatment of siloxanes with such toxic agents as various amines [13, 14]; thermal degradation (300–400 °C); and treatment with sulfuric acids, thionyl chloride, and mixtures of alkali (NaOH, KOH) or with alcohols (methanol, ethanol) [15–18]. In our previous work, we found that dimethyl carbonate, which is an environmentally friendly reagent [19, 20] that meets all the requirements of green chemistry, promotes partial depolymerization of organosiloxanes, making the resultant oligomers a candidate for surface functionalization [21]. However, no systematic characterization on the surface species of various depolymerized organosiloxanes on silica surface has been performed. Useful but limited information on the bonded species of silylated silica surfaces can be obtained through zeta potential, infrared spectroscopy, scanning, and transmission electron microscopy. One of the problems often met with these methods concern the difficulty in the identification of different OH and Si–O bonds. More specific information can be obtained by high-resolution ^13^C and ^29^Si cross-polarization magic-angle spinning NMR (CP-MAS NMR) and ^1^H MAS NMR. Indeed, only the use of abovementioned method allows a full characterization of the surface species on silylated silica. Some solid-state NMR studies have been already performed on gel and fumed silicas modified with different alkoxysilanes [22–28], mesoporous silica modified with cetyltrimethylammonium bromide [29], and 3-metacryloxypropyltrimethoxysilane (MPS) deposited in various solvents onto porous silica [30].

Therefore, the aim of this work is to study the surface species of various organosiloxanes and their mixtures with dimethyl or diethyl carbonate at a fumed silica surface depending on the polymer chain length of siloxane used as a modifying agent and on the nature of dimethyl or diethyl carbonate applied as an initiator for partial organosiloxane deoligomerization.

## Experimental Methods

### Chemical Reagents

For preparation of the modified silica surfaces, poly(methylhydrosiloxane) (code name PMHS, linear, –CH_3_ terminated, viscosity of *ca*. 3 cSt at 25 °C), poly(dimethylsiloxane) (code name PDMS, linear, –CH_3_ terminated, viscosity of *ca*. 100 cSt at 25 °C), and poly[dimethylsiloxane-*co*-(2-(3,4-epoxycyclohexyl)ethyl)methylsiloxane] (code name CPDMS, linear, –CH_3_ terminated, viscosity of *ca*. 3,300 cSt at 25 °C) were purchased from Sigma Aldrich, USA. Commercial, dimethyl carbonate (DMC), diethyl carbonate (DEC), and fumed silica (SiO_2_, *S*_BET_ = 278 m^2^/g) were purchased from Aladdin Reagents, China. The purity of the reagents, as reported by the manufacturers, was ≥ 99.0 %. The reagents were used as received.

### Modification of Fumed Silica Surfaces

Organosiloxanes were chosen as non-toxic and environmentally benign modifying reagents with high carbon content. FSNs were applied as a matrix for modification because of the high regularity hydroxyl groups on the surface and good dispersibility. In addition, the main advantage of these FSNs over larger monodisperse particles is the fact that they provide a large surface area and thus high sensitivity for solid-state NMR. The modification of the fumed silica surface was performed with PMHS, PDMS, and CPDMS at 180–200 °C for 2 h with or without addition of DMC or DEC, which does not contribute to the weight of modified silica due to the reaction mechanism in gaseous (nitrogen) dispersion media (i.e., without a solvent). The amount of modifier agent was determined to be 17 wt% of silica weight. The modification process was performed in a glass reactor with a stirrer with a rotational speed of 350–500 rpm. The modifying agent was added by means of aerosol-nozzle spray. The samples were subsequently cooled to room temperature after the synthesis.

### Elemental Analysis

The content of grafted organic groups in the synthesized samples was measured a couple of times by a vario MACRO cube analyzer (Elementar, Germany), and average values for carbon content and relative deviations were calculated (Table 1). The anchored layer was oxidized to produce H_2_O and CO_2_ during heating of the samples in the oxygen flow at 750 °C.

**Table 1:** Carbon content, bonding density, and surface area of grafted neat organosiloxanes and their mixtures with DMC or DEC at the SiO_2_ surface

Sample	Carbon content, wt%	Bonding density ([Si(CH_3_R_1_O)), groups/nm^2^ where R_1_ = CH_3_, H, CH_2_CH_2_C_6_H_9_	*S*_BET_, m^2^/g
SiO_2_ (A–300)	0	0	278
SiO_2_/PMHS	2.42 ± 0.08	5.5	266
SiO_2_/PMHS+DMC	2.60 ± 0.04	6.0	253
SiO_2_/PMHS+DEC	2.17 ± 0.11	5.0	254
SiO_2_/PDMS	5.96 ± 1.17	7.2	220
SiO_2_/PDMS+DMC	6.04 ± 0.01	7.4	167
SiO_2_/PDMS+DEC	2.28 ± 0.20	2.5	235
SiO_2_/CPDMS	0.57 ± 0.04	0.1	274
SiO_2_/CPDMS+DMC	1.77 ± 0.01	0.4	248
SiO_2_/CPDMS+DEC	2.98 ± 0.28	0.7	261

The bonding density of the attached layers was calculated using the formula [11, 12]

1 $$ \rho =\frac{6\times {10}^5\left(\%C\right)}{\left[1200\times {n}_c-{M}_w\times \left(\%C\right)\right]}\frac{1}{S\left(\mathrm{BET}\right)}, $$

where *M*_*w*_ is the molecular weight of the grafted group, %*C* is the carbon weight percentage of the modified silica, *S*(BET) is the surface area of the original silica (m^2^/g), and *n*_*с*_ is the number of carbon atoms in the grafted group in each silicone used for modification. Equation 1 gives the number of [–Si(RR_1_)O–] repeat units per 1 nm^2^ of the surface (*ρ*) (where *R* is methyl group (CH_3_); *R*1 is hydro (H) or methyl (CH_3_) or epoxy(cyclohexylethyl) group (CH_2_CH_2_C_6_H_9_)).

### ^29^Si, ^1^H, and ^13^C CP/MAS NMR Measurements

Solid-state ^1^H MAS NMR spectra were recorded on a Bruker Avance 400 III HD spectrometer (Bruker, USA, magnetic field strength of 9.3947 T) at resonance frequency of 79.49 MHz. The powder samples were placed in a pencil-type zirconia rotor of 4.0 mm o.d. The spectra were obtained at a spinning speed of 10 kHz, with a recycle delay of 1 s. The adamantane was used as the reference of ^1^H chemical shift.

Solid-state ^29^Si CP/MAS NMR spectra were recorded on a Bruker Avance 400 III HD spectrometer (Bruker, USA, magnetic field strength of 9.3947 T) at resonance frequency of 79.49 MHz for ^29^Si using the cross-polarization (CP), magic-angle spinning (MAS), and a high-power ^1^H decoupling. The powder samples were placed in a pencil-type zirconia rotor of 4.0 mm o.d. The spectra were obtained at a spinning speed of 8 kHz (4 *μ*s 90° pulses), a 8-ms CP pulse, and a recycle delay of 4 s. The Si signal of tetramethylsilane (TMS) at 0 ppm was used as the reference of ^29^Si chemical shift.

Solid-state ^13^C CP/MAS NMR spectra were recorded on a spectrometer (Bruker, USA, with a magic field strength of 9.3947 T) at a resonance frequency of 100.61 MHz for ^13^C using the cross-polarization (CP), magic-angle spinning (MAS), and a high-power ^1^H decoupling. The powder samples were placed in a pencil-type zirconia rotor of 4.0 mm o.d. The spectra were obtained at a spinning speed of 5 kHz (4 *μ*s 90° pulses), a 2-ms CP pulse, and a recycle delay of 4 s. The methylene signal of glycine at 176.03 ppm was used as the reference of ^13^C chemical shift.

### ^1^H Liquid NMR Spectroscopy

^1^H NMR spectra of each initial organosiloxane (PMHS (Additional file 1: Figure S1), PDMS (Additional file 1: Figure S2), CPDMS (Additional file 1: Figure S3); see Additional file 1) were recorded at 90 MHz with an Anasazi Eft–90 spectrometer (Anasazi Instruments, USA). Each polymer was dissolved in deuterated chloroform CDCl_3,_ and the resulting solution was analyzed by ^1^H NMR spectroscopy.

### BET Measurements

To analyze the surface area (*S*_BET_, m^2^/g) of the silicas, the samples were degassed at 150 °C for 300 min. Low-temperature (77.4 K) nitrogen adsorption–desorption isotherms were recorded using a Micromeritics ASAP 2420 adsorption analyzer (Micromeritics Instrument Corp., USA). The specific surface area (Table 1, *S*_BET_) was calculated according to the standard BET method.

## Results and Discussion

**Fig. 1:**
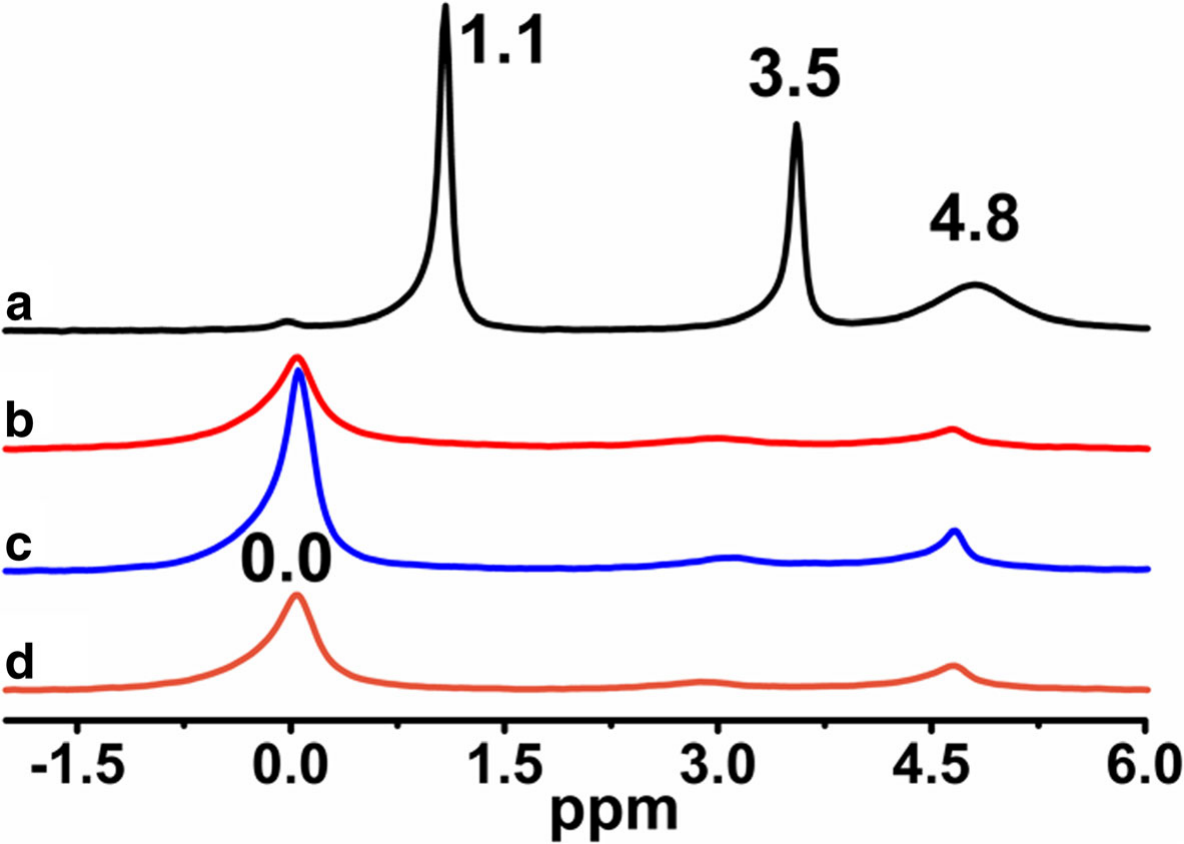
^1^H MAS NMR spectra of (**a**) neat fumed silica, (**b**) modified fumed silica with PMHS, modified with (**c**) mixtures of PMHS and dimethyl carbonate and (**d**) mixtures of PMHS and diethyl carbonate

**Fig. 2:**
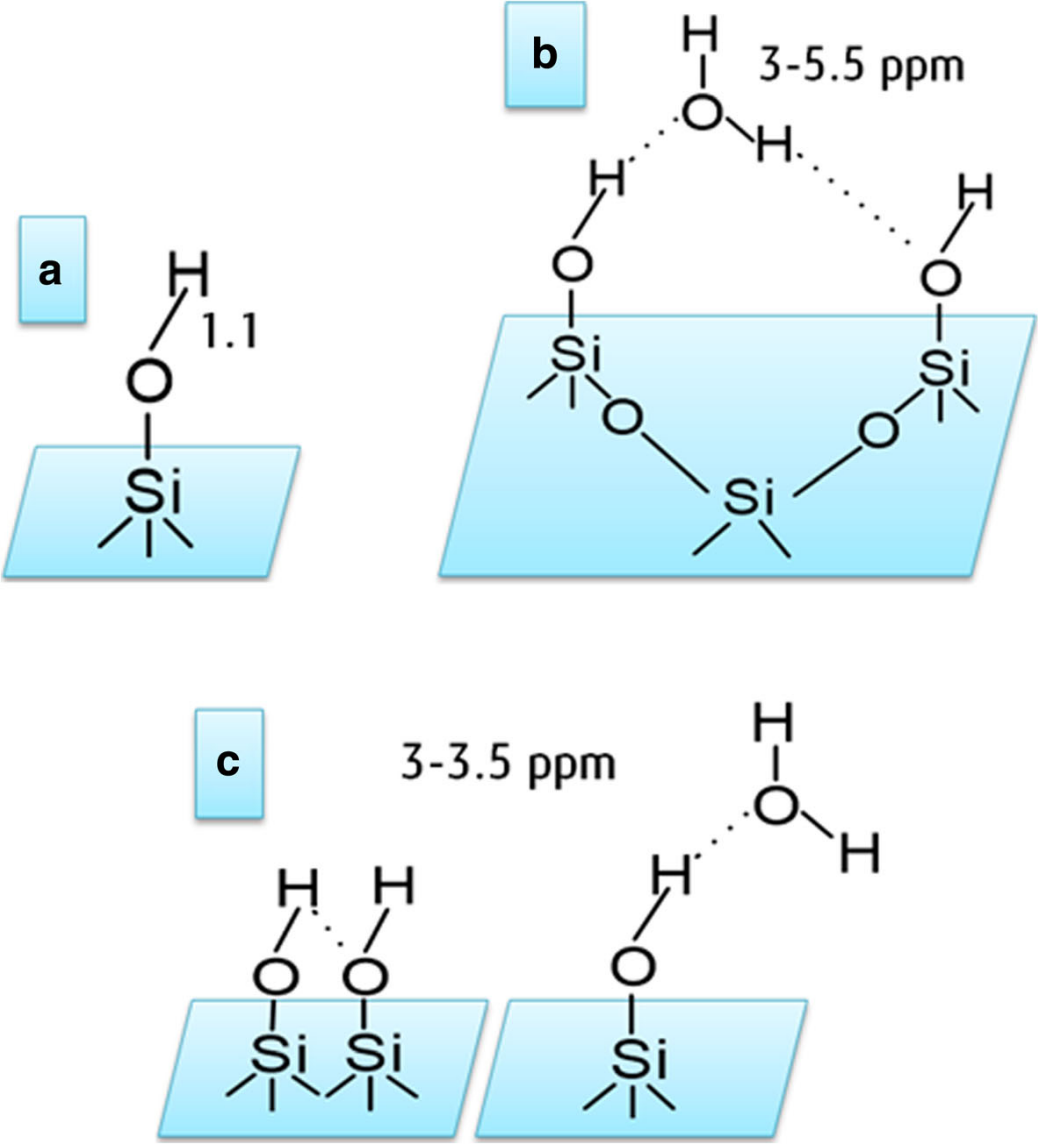
Schematic representation of (**a**) single silanol groups and (**b**, **c**) possible structures involving the silanol groups and physisorbed water at the silica surface. The values of chemical shift are assigned to these structures according to refs [26, 27, 29]

^1^H MAS NMR spectrum of neat fumed silica, Fig. 1 a, represents three main contributing peak lines at 1.1, 3.5, and 4.8 ppm. The peak at 1.1 ppm is assigned to isolated silanols at the SiO_2_ surface. Note that they were not detected directly at their usual position around 1.8 ppm in the spectrum of neat silica, but it is known that protons in isolated silanol groups also produce lines between 0.5 and 1.5 ppm. Similar spectral features were also observed in previous studies [29]. Chemical-shift lines between 3.5–5.0 ppm are assigned to weakly bound, relatively mobile water, and hydrogen bonded silanols (Figs. 1 and 2) [26, 27, 29].

The intense resonance in the 3.5–5.0 ppm range has been studied widely by different researchers. Liu and Maciel [26], for example, by using CRAMPS observed a peak at 4.1 ppm in humidified fumed silica (Cab–O–Sil) which they reported as intermediate between that of liquid water protons (4.9 ppm) and that of the physisorbed water peak (3.5 ppm). According to their studies, a resonance at 3.5 ppm assigned to physisorbed water could easily be desorbed by evacuation at 25 °C. Moreover, evacuation at 100 or 225 °C led to further decrease in the intensity of this resonance, and it was attributed to “rapidly exchanging weakly hydrogen bonded hydroxyls, including those of both water and silanols” [25, 29]. On the other hand, the ^1^H MAS NMR investigation of silicas by Turov et al. [31, 32] reported the chemical shift of water at around 5 ppm at 25 °C. Several other studies of silicas have also attributed the resonances at 4.5–5.0 ppm to water on strongly hydrated surfaces and chemical shift near 3 ppm to water on significantly dehydrated surfaces, as reported by Turov et al. [32].

**Fig. 3:**
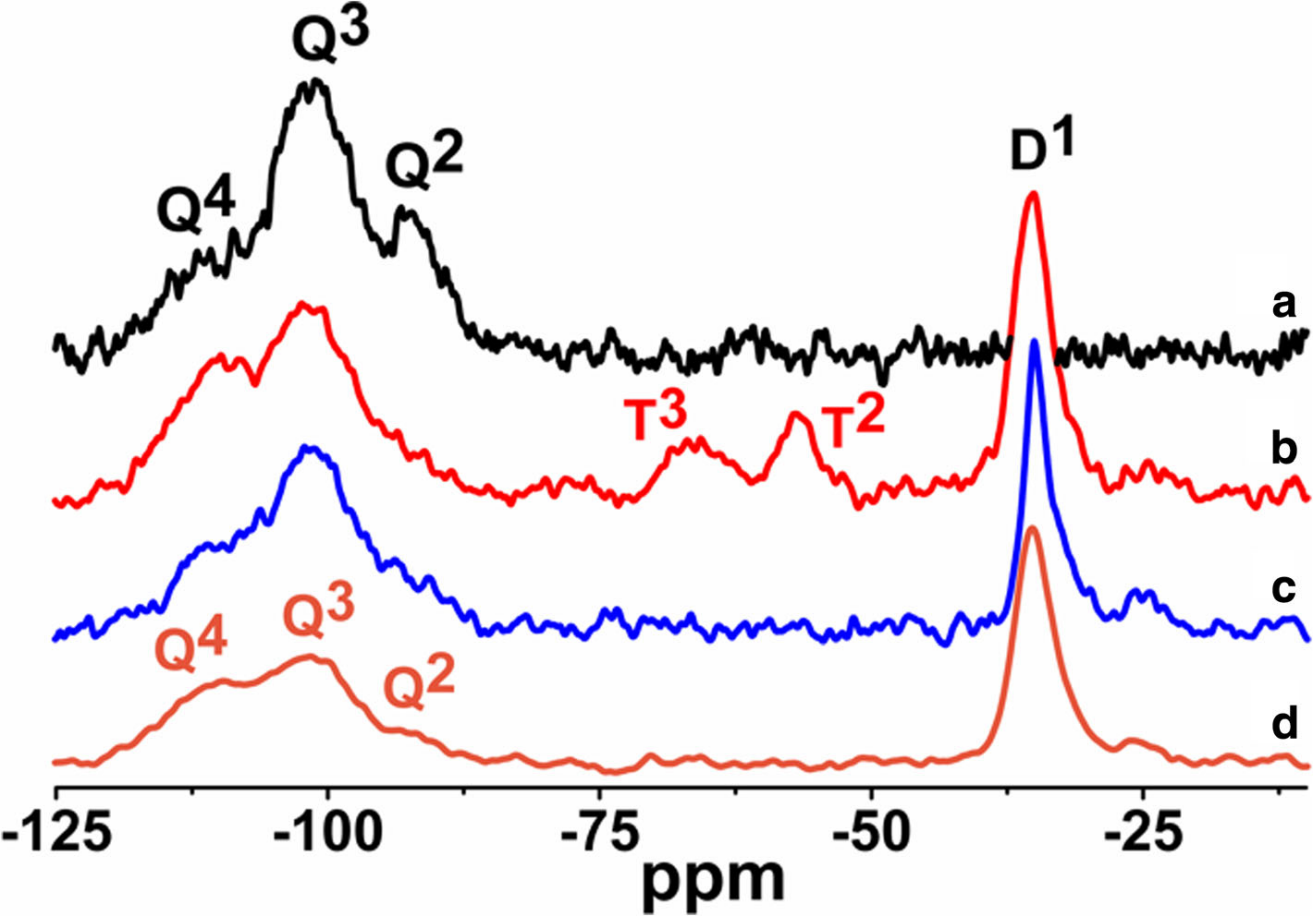
^29^Si CP/MAS NMR spectra of (**a**) neat fumed silica, (**b**) modified fumed silica with neat PMHS, modified with (**c**) mixtures of PMHS and dimethyl carbonate and (**d**) mixtures of PMHS and diethyl carbonate

**Fig. 4:**
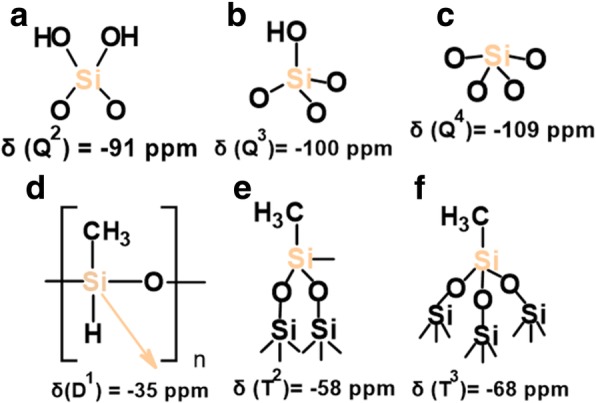
Various grafted PHMS species (**a**–**f**)

The ^1^H MAS NMR spectra of modified silicas (Fig. 1) with neat poly(methylhydro)siloxane (curve b) and its mixture of DMC or DEC (curves c and d) were similar to each other and displayed a broad peak (centered at 0.0 ppm) which confirms the grafting of alkyl siloxane species. All the spectra of presented samples show the intensity reduction of adsorbed water and hydrogen bonded silanols (3.5–5.0 ppm) and do not show the isolated silanols (1.1 ppm) presence, confirming that silicas were well modified. The appearance of the peak around 4.7 ppm can be assigned also to the proton in the Si-H group which was attached to the SiO_2_ surface, as well as alkyls during SiO_2_ functionalization. The presence of the adsorbed water in the modified samples can be explained by the fact that water molecules are much smaller than the cross-section of organosiloxane. Water can therefore penetrate the narrow nanovoids in the contact zones between adjacent nanoparticles in the aggregates, but polymer macromolecules cannot penetrate these voids. Nevertheless, the ^1^H MAS NMR spectra of modified silicas provide much less structural information than the ^29^Si CP/MAS NMR spectra of these composites in which is possible to see unique resonances of different grafted species [33]. The nomenclature used to define siloxane surface species grafted at the silica surface incorporates the use of the letters M, D, T, and Q of the organosiloxane units which represent R_3_SiO_0.5_, R_2_Si(O_0.5_)_2_, RSi(O_0.5_)_3_, and Si(O_0,5_)_4_ units, respectively, where *R* represents aliphatic and/or aromatic substituents or H [34]. The CP/MAS ^29^Si NMR spectrum of unreacted silica (Fig. 3 a) shows three signals with resolved peaks at − 91, − 100, and − 109 ppm. These peaks are assigned to silicon atoms in the silanediol groups, silanol groups, and silicon-oxygen tetrahedra of the SiO_2_ framework, respectively (Fig. 4a–c and Table 2), or, in other words, to silicon-oxygen tetrahedra Q^2^, Q^3^, and Q^4^ where the superscript indicates the number of siloxane bonds [34]. Appropriately, an assignment was made on the basis of the small difference between ^29^Si chemical shifts in solids and the corresponding shifts in a liquid, and data on soluble silicates were used for identification. The formation of an additional siloxane bond has been found to lead to an upfield signal shift of about 9 ppm [28].

**Table 2:** ^29^Si Chemical shifts (*δ*) of grafted neat and depolymerizied organosiloxane species at SiO_2_ surface

Species	*δ*, ppm
Si(OH)_2_(O–)_2_	− 91
Si(OH)(O–)_3_	− 100
Si(O–)_4_	− 109
Si(CH_3_)(H)(O–)	− 35
(≡SiO)_2_SiR, R-attached polymer chain	− 58
(≡SiO)_3_SiR, R-attached polymer chain	− 68
Si(CH_3_)(R)(O–)_2_ in D_4_, R = CH_3_, C_2_H_4_C_6_H_8_	− 19.5
Si(CH_3_)(O–)_2_ in linear MD_4_M	− 23
Si(CH_3_)_2_(O–)_2_, D_1_	− 21
Si(CH_3_)(C_2_H_4_C_6_H_8_), D_2_	− 23

As can be seen in Fig. 3, after silica surface modification with low viscous poly(methylhydrosiloxane) and DEC (curve d), a significant decrease in the signals Q^3^ and Q^2^ is accompanied by an increase in the intensity growth of the signal Q^4^. Additionally, the signal at − 35 ppm appears, and this can be identified with the methylhydrosiloxane species (D^1^), (Fig. 4d and Table 2). This implies that a reaction has occurred between the silica surface and the PHMS/DEC mixture.

**Fig. 5:**
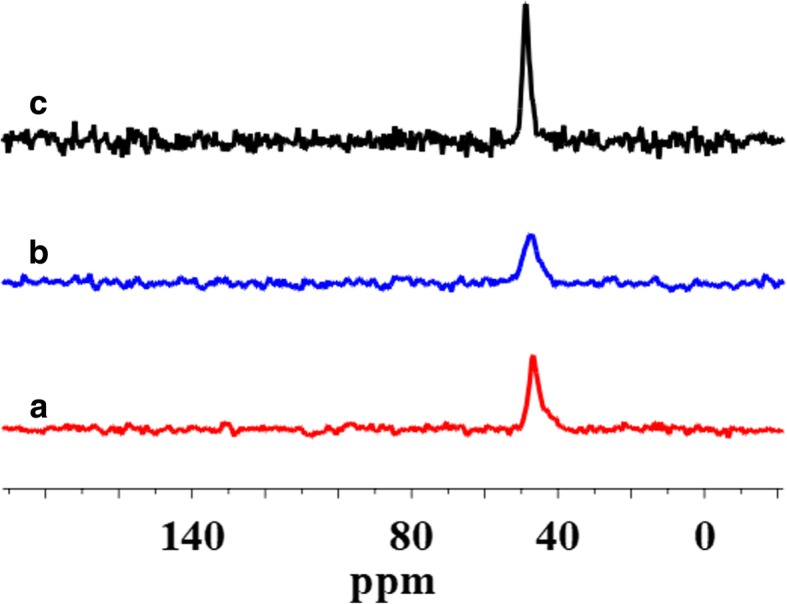
^13^C CP/MAS NMR spectra of (**a**) modified fumed silica with neat PMHS, modified with (**b**) mixtures of PMHS and dimethyl carbonate and (**c**) mixtures of PMHS and diethyl carbonate

The surface of SiO_2_/PMHS+DEC also shows high grafting density of 5.0 groups/nm^2^ (Table 1) and larger diminution of *S*_BET_ (Table 1) as compared with SiO_2_ modified with neat PMHS suggesting closely packed methylhydrosiloxane network. Close values of grafting densities have been reported for the self-assembled monolayers (SAMs) of C_18_H_37_SiH_3_, C_18_H_37_SiCl_3_, and C_18_H_37_P(O)(OH)_2_ on metals and metal oxides [35–37] and C_18_H_37_SH on gold [38]. The silicas modified with neat PMHS and its mixture of DMC show a grafting density even slightly higher around 5.5–6.0 groups/nm^2^ (Table 1). Nevertheless, the appearance of the chemical shifts at − 35, − 58, − 68 ppm of the D^1^, T^2^, and T^3^ units (Fig. 4d–f) for SiO_2_/PMHS (Fig. 3 b) and only the D^1^ unit for SiO_2_/PMHS+DMC (Fig. 3 c) is not accompanied by a significant reduction of the peak which corresponds to free silanols (− 100 ppm, Q^3^) as is the case for SiO_2_/PMHS+DEC (Fig. 3 d). The ^13^C CP/MAS NMR spectra of these modified FSNs (Fig. 5) show one prominent peak at about 43–46 ppm due siloxane alkyl chains grafted at their SiO_2_ surfaces. The sharp peak in the CP/MAS ^13^C NMR spectrum of SiO_2_/PMHS+DMC (Fig. 5 c) indicates well-ordered surface structures at the SiO_2_ surface. On the contrary, in the CP/MAS ^13^C NMR spectra of SiO_2_/PMHS (Fig. 5 a) and SiO_2_/PMHS+DMC (Fig. 5 b), the signals are relatively broad, indicating a restricted mobility of the functional groups attached to the siloxane framework. Additionally, a higher relative intensity of this signal 43–46 ppm for SiO_2_/PMHS+DEC may suggest a greater number of attached surface species at the SiO_2_ surface as compared with SiO_2_/PMHS and SiO_2_/PMHS+DMC.

**Scheme 1:**
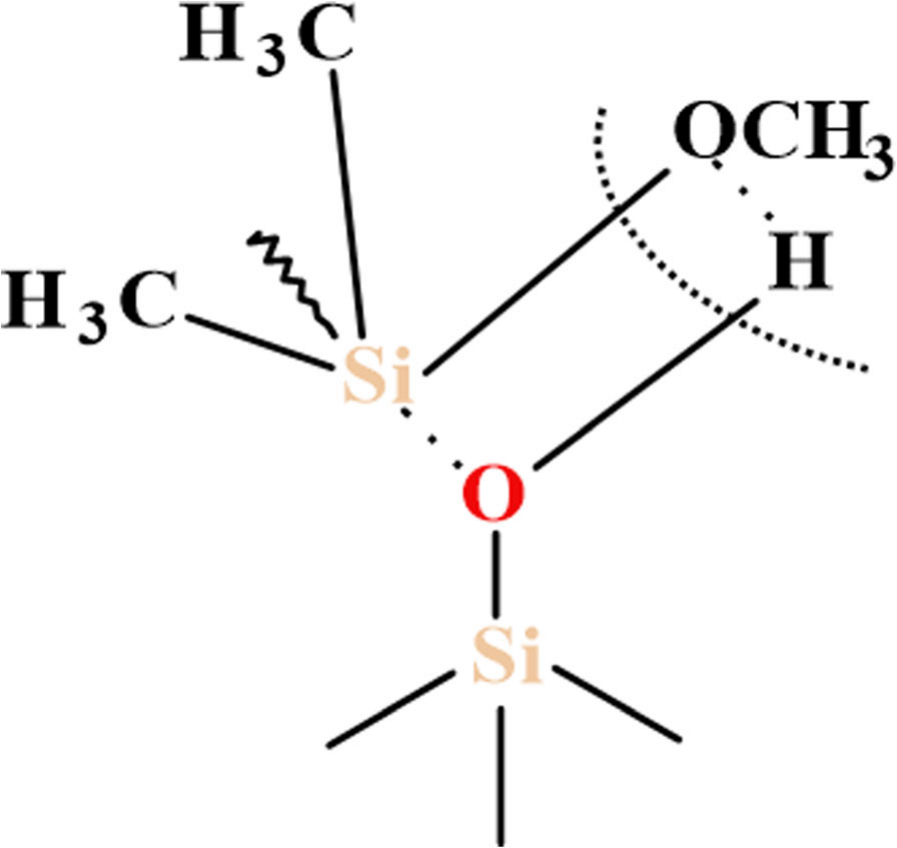
Attack by methoxysilane of silica silanol group

**Fig. 6:**
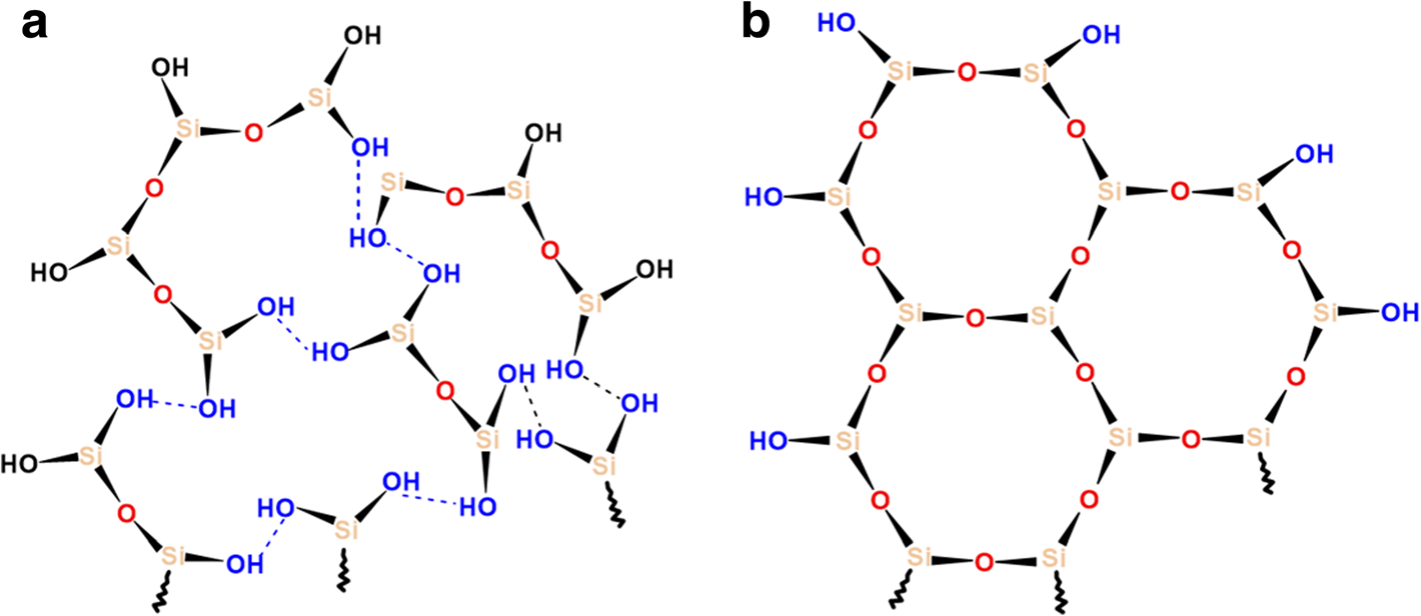
The amorphous-like structure (**a**) consists of the molecules bonded via Si-O-Si and Si-OH·····HO-Si bonds, proposed in [11] and (**b**) the crystalline-like structure has “extended” Si-O-Si bonds, proposed in [11, 38–41]

The abovementioned differences of modified silicas could be explained by several factors: (1) a type of organosiloxane bonding (physical or chemical) with SiO_2_ surface and (2) changes in the length of initial organosiloxane and its fragments after reactions with alkyl carbonate (as shorter polymer fragments will react intensively with silica surface sites due to the lower of steric hindrance of side polymer chains). It is therefore more likely that neat PHMS and the mixture of PHMS/DMC absorb at the SiO_2_ surface through the formation of adsorption complexes by the binding of hydrogen in the surface silanol group with siloxane oxygen of organosiloxane, while the chemical reaction between the SiO_2_ surface and PMHS/DEC could be carried out through the formation of chemical bond by the electrophilic substitution of the proton in the silanol group (see Scheme 1 below). The latter explains the significant reduction of free silanols peak at − 100 ppm (Q^3^) for SiO_2_/PHMS+DEC (Fig. 3 d). That fact that the resonance of isolated silanols (-100 ppm) is significantly decreased for SiO_2_/PHMS+DEC in comparison to unreacted SiO_2_ but does not disappear completely (even with close packing of methylhyrosiloxane groups of 4.0 group/nm^2^) indicates that some of the OH groups were inaccessible to the modifier reagent. These silanols could be located inside SiO_2_ nanoparticles. Note that these intra-particle silanols and water molecules can be removed upon heating at 550–700 °C, and only a very small amount of residual silanols remains upon heating even at 1000 °C [11]. The existence of intracrystalline hydroxyl groups is typical for layered silicates [28]. According to Iler [1], their formation is possible in an aerosil structure by the aggregation of SiO_2_ primary particles with a size of 1–2 nm into a finite globule with a diameter of 10–20 nm. In addition, one cannot rule out the possibility of internal hydroxy group formation in the course of diffusion of the water molecules into the SiO_2_ globules. On the other hand, unreacted silanols play an important role in the stabilization of alkylsilanes layers at the SiO_2_ surface as considered by other researchers [11, 35–43]. In the opinion of the authors [11], grafted silane layers form a closely packed monolayer film with an ordered amorphous structure with a significant number of the uncoupled silanols that interact with neighboring Si–OH groups via hydrogen bonding, while the alkyl chains (not shown in Fig. 6) are directed perpendicular to the plane of the siloxane network (Fig. 6a). The presence of uncoupled silanols supports enough space for the presence of alkyl chains grafted at SiO_2_ after the modification, as the maximal length of the Si–OH·····HO–Si sequence of bonds is ≈ 0.6 nm, which is notably higher than the Van de Waals diameter of the alkyl chains (≈ 0.46 nm). In the case of the absence of the uncoupled silanols, the attached monolayers form a hexagonal array (Fig. 6b) where Si atoms are connected via the siloxane network [39]. However, as was reported by Helmy et al. [11], such a structure is too constrained by steric repulsion between the grafted alkyl chains, as the maximum length of Si–O–Si bond is 0.32 nm, which is very much smaller than the Van der Waals diameter of the alkyl chain (0.46 nm).

**Fig. 7:**
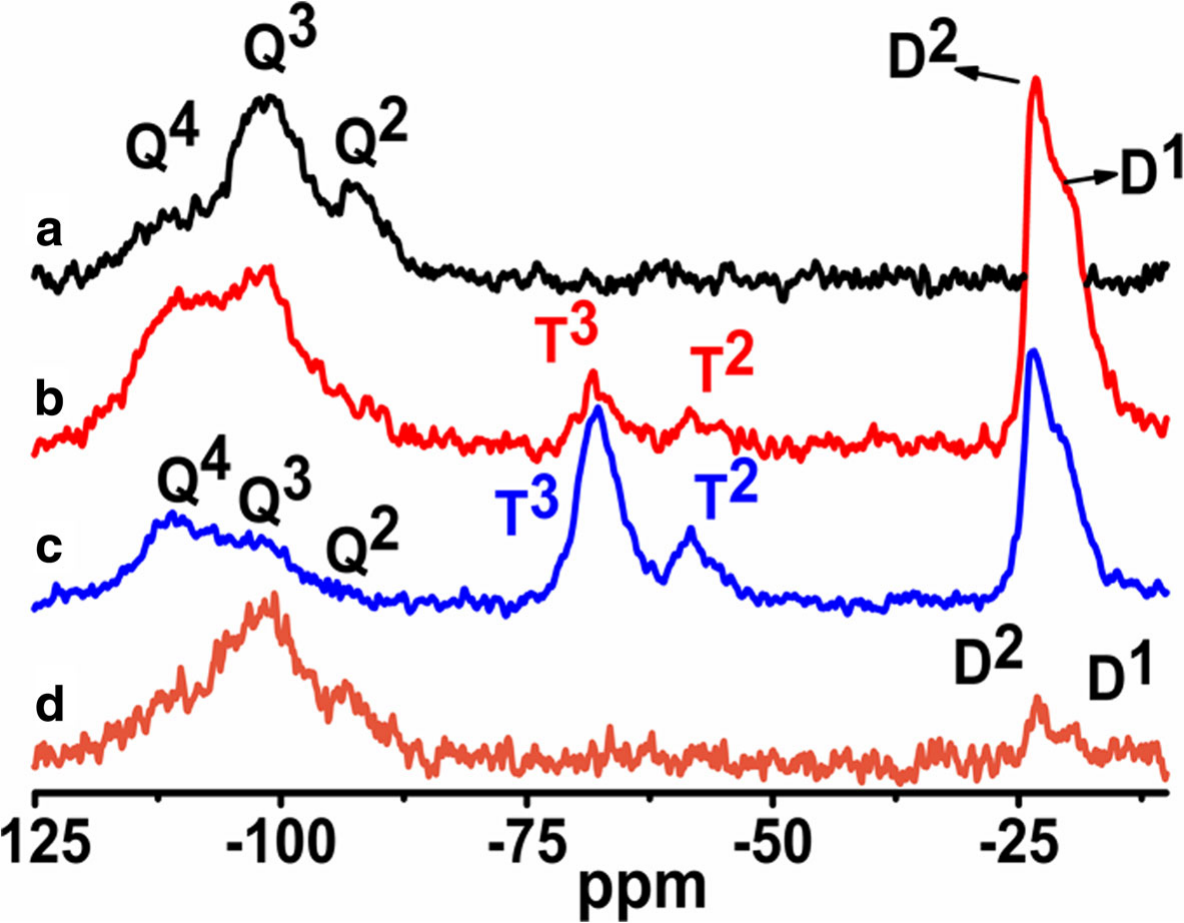
^29^Si CP/MAS NMR spectra of (**a**) neat fumed silica, (**b**) modified fumed silica with PDMS, modified with (**c**) mixtures of PDMS and dimethyl carbonate and (**d**) mixtures of PDMS and diethyl carbonate

**Fig. 8:**
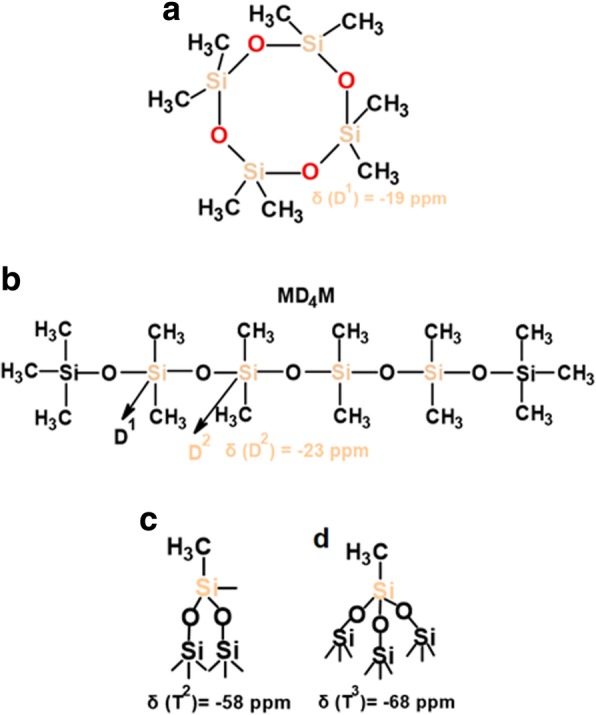
Various grafted PDMS species (**a**–**d**)

**Fig. 9:**
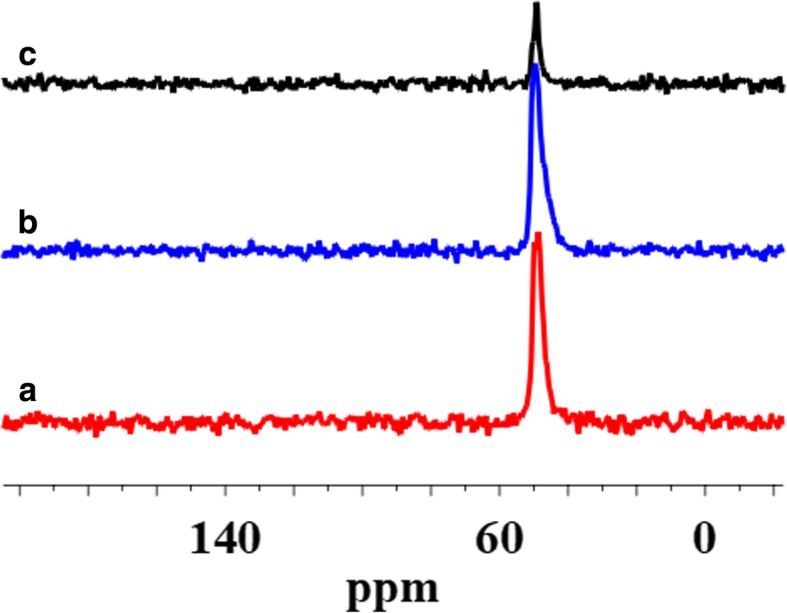
^13^C CP/MAS NMR spectra of (**a**) modified fumed silica with neat PDMS, modified with (**b**) mixtures of PDMS and dimethyl carbonate and (**c**) mixtures of PDMS and diethyl carbonate

^29^Si CP/MAS NMR spectra of SiO_2_ modified with organosiloxane of medium chain length (PDMS) and its mixture with DMC or DEC are shown in Fig. 7. The chemical shifts, which appeared at − 19 and − 23 ppm for all the samples (Table 2), are assigned to D^1^ and D^2^ units in cyclotetrasiloxane and dimethylsiloxane species in linear MD_4_M, respectively (Fig. 8 a, b). Notice that the shift of dimethylsiloxane species (− 23 ppm) for silicas modified with PDMS (Fig. 7) is shifted to higher frequency ranges in comparison to SiO_2_ modified with PMHS (− 35 ppm, Fig. 3), which is explained by the fact that hydrides appear at relatively low frequency compared with their alkyl analogs [34]. The abovementioned resonances result from “capping” of the silica surfaces with modifier agent which is in a good agreement with earlier reports [5]. The sites denoted as T^2^ and T^3^, observed around − 58 and − 68 ppm for SiO_2_/PDMS and SiO_2_/PDMS+DMC (Fig. 7 b, c) are assigned to (≡SiO)_2_SiR and (≡SiO)_3_SiR functionalities (Fig. 8 c, d) where *R* represents the attached polymer chain. The presence of D as well as T sites for these samples indicates that functionalization of the SiO_2_ surfaces has occurred. Note that the appearance of the chemical shifts of the D and T units for SiO_2_ modified with mixture of PDMS/DMC (Fig. 7 c) is accompanied by a significant decrease in the resonances of free and geminal silanols and also *S*_BET_ value (167 m^2^/g, Table 1), which may suggest that the reaction of the SiO_2_ with depolymerized PDMS occurred through the chemical bonding, as for SiO_2_/PMHS+DEC (Fig. 3 d). The surface also shows the highest grafting density (*ρ*_max_) – 7.4 groups/nm^2^ and the lowest surface area in comparison with other silicas presented in this work (Table 1). The *ρ*_max_ value obtained for SiO_2_/PDMS+DMC is similar to those reported for the best quality monolayers derived from chloro- and aminosilanes [35, 44]. The only difference is that the modification of SiO_2_ with mixture of PDMS/DMC occurs with noncorrosive reagents, thus providing a cleaner and less hazardous environment than amino- and chlorosilanes. The high value of the grafting density for this surface indicates the formation of closely packed grafted organic layers. On the contrary, the resonances of free and geminal silanols are not shown to be greatly diminished in intensity for the surfaces, which were obtained by SiO_2_ modification with neat PDMS (Fig. 7 b) and its mixture of DEC (Fig. 7 d) but showing grafting density 7.2 and 2.5 groups/nm^2^. This could be explained by the partial adsorption of the modifier reagent at the SiO_2_ surface as was mentioned in the previous section. ^29^Si CP/MAS NMR data are in a good agreement with the BET data (Table 1), as surface areas for both samples are higher compared with SiO_2_/PDMS+DMC, confirming the smaller degree of chemisorption of the modifier agents at the SiO_2_ surfaces. In addition, the ^13^C CP/MAS NMR data (Fig. 9) are in excellent agreement with the data on grafting density (Table 1) and ^29^Si CP/MAS NMR data, and the relative intensities of the signals which correspond to organosiloxane chains (44–50 ppm, Fig. 9) attached to the SiO_2_ surface are higher for SiO_2_/PDMS+DMC (curve b) and SiO_2_/PDMS (curve a) as compared with SiO_2_/PDMS+DEC (curve c). All the signals in the ^13^C CP/MAS NMR spectra (Fig. 9) are relatively sharp, indicating well-ordered surface structures on the silica surface.

**Scheme 2:**
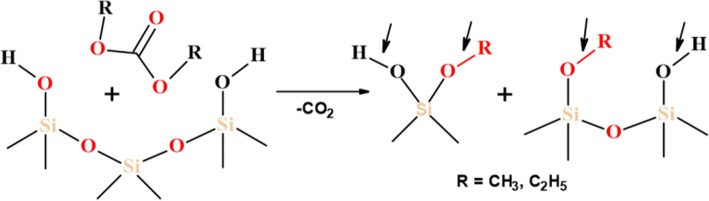
The reaction of DMC or DEC with the SiO_2_ surface

The denser coverage for SiO_2_/PMHS+DEC (discussed above) and SiO_2_/PDMS+DMC in comparison to other samples presented here can be explained also by the presence of additional reactive centers at the SiO_2_ surface, the attached methoxy groups (–OCH_3_ or OR), which can be formed by the reaction of DMC or DEC with the SiO_2_ surface (see Scheme 2 below).

**Fig. 10:**
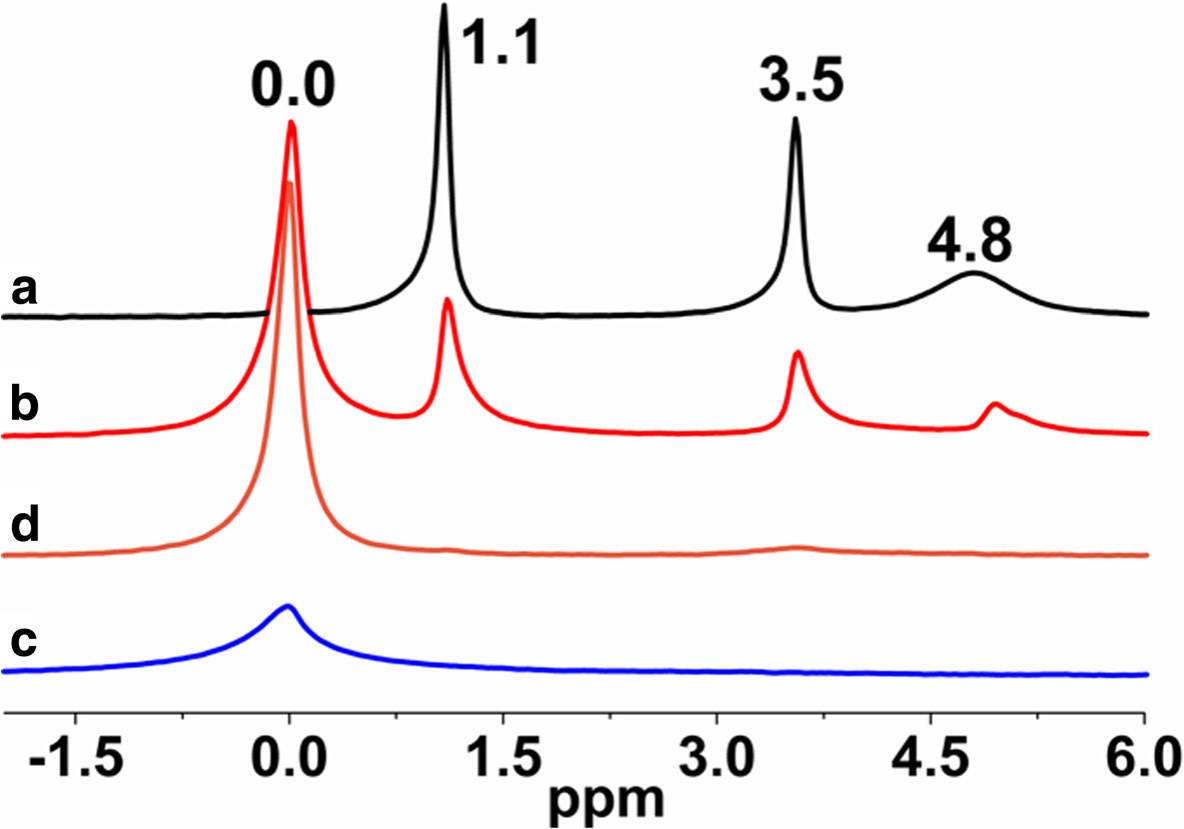
^1^H MAS NMR spectra of (**a**) neat fumed silica, (**b**) modified fumed silica with PDMS, modified with (**c**) mixtures of PDMS and dimethyl carbonate and (**d**) mixtures of PDMS and diethyl carbonate

^1^H MAS NMR spectra of FNSs modified with mixtures of PDMS/DMC or PDMS/DEC (Fig. 10 c, d) show the disappearance of the peaks of free and hydrogen-bonded silanols (*δ* = 1.1 ppm and *δ* = 4.8 ppm) as well as adsorbed water (*δ* = 3.5 ppm). The presence of grafted siloxane species is confirmed by the emergence of a chemical shift at 0.0 ppm for all the samples (Fig. 10 b–d). In spite of the methylsiloxane grafting presence for SiO_2_/PDMS (Fig. 10 b), its surface still contains free, hydrogen-bonded silanols and adsorbed water, which is in very good agreement with ^29^Si CP/MAS NMR data (Fig. 7 b). The values of the bonding density (Table 1) of the attached layers of FSNs modified with short PMHS and its mixture of DMC or DEC, as well as medium PDMS and its mixture of DMC, suggest a “vertical” orientation of the grafted dimethylsiloxane and methylhydrosiloxane molecules stabilized by lateral Si–O–Si bonds and Van der Waals interactions between the grafted alkyl chains [43–46], while FSNs modified with PDMS/DEC mixture show a grafting density of 1.9 groups/nm^2^, suggesting a “horizontal” orientation of the grafted dimethylsiloxane molecules [8, 35].

Overall, from the solid-state NMR data obtained, it is evident that the addition of DMC to the modifying mixture has a significant effect on the chemical interaction of organosiloxane of a medium length of polymer chain (PDMS) used for modification at the silica surface, while DEC addition has practically no influence on the chemical interaction of SiO_2_ with PDMS. In contrast, the DEC has a great effect on the chemical interaction of short organosiloxane (PMHS) used for modification at the SiO_2_ surface, while DMC has minimal impact on the chemical interaction of SiO_2_ with PMHS.

**Fig. 11:**
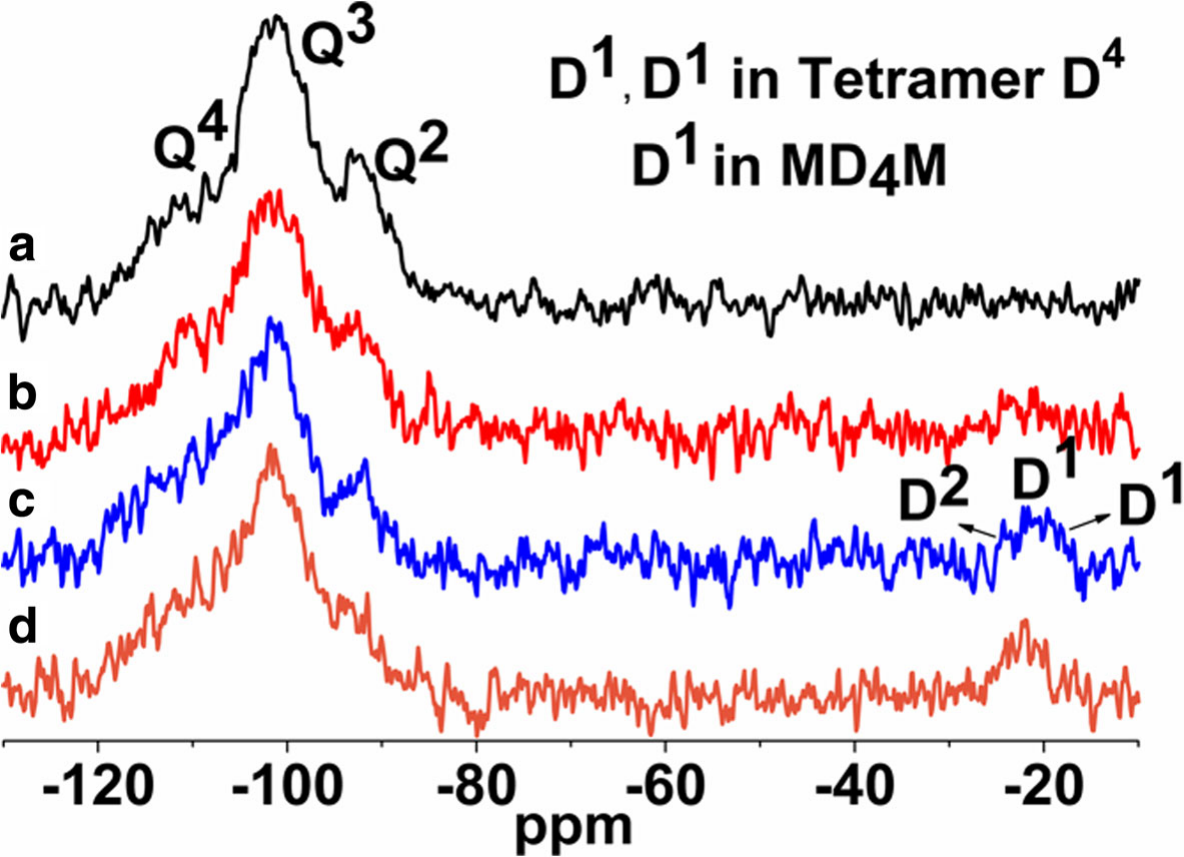
^29^Si CP/MAS NMR spectra of (**a**) neat fumed silica, (**b**) modified fumed silica with CPDMS, modified with (**c**) mixtures of CPDMS and dimethyl carbonate and (**d**) mixtures of CPDMS and diethyl carbonate

**Fig. 12:**
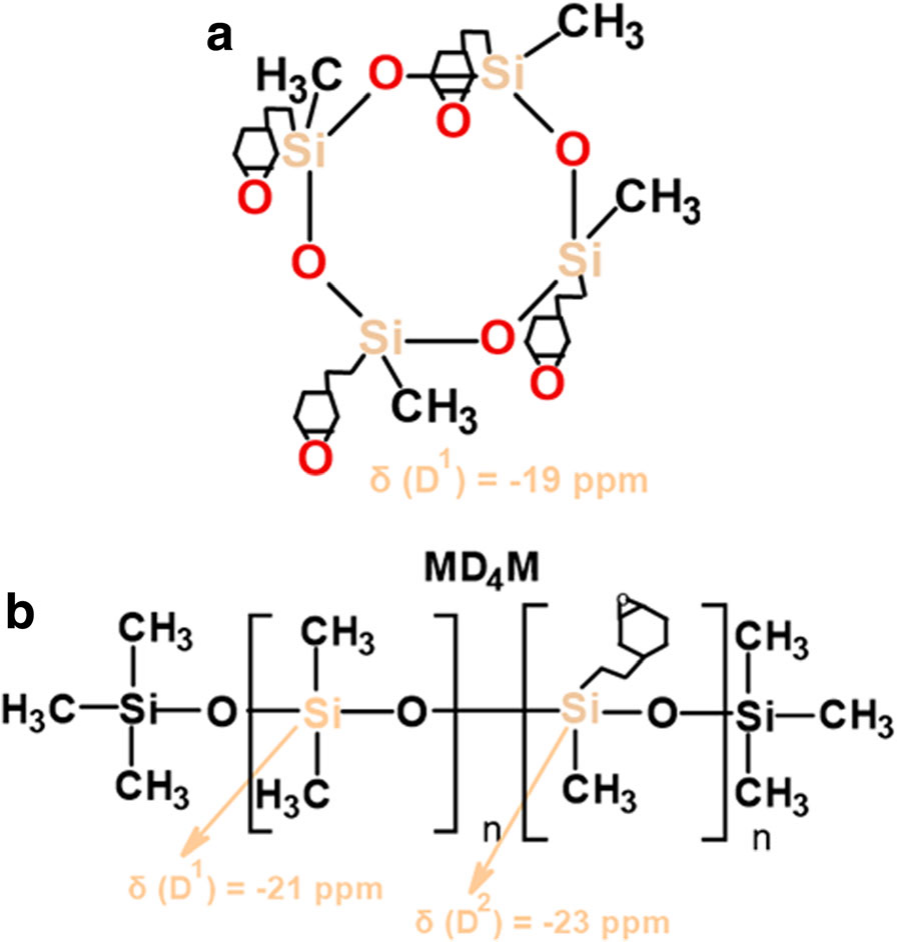
Various grafted CPDMS species (**a**, **b**)

As can be seen from the ^29^Si CP/MAS NMR spectrum of SiO_2_ modified with the longest polymer, poly[dimethylsiloxane-co-(2-(3,4-epoxycyclohexyl)ethyl)methylsiloxane (CPDMS) (Fig. 11 a), the resonances of grafted methyl-epoxy species around − 23 and − 19 ppm are very hardly detectable, which implies mostly an inert nature of this polymer in relation to the SiO_2_ surface. ^29^Si CP/MAS NMR spectra of silicas modified with the longest organosiloxane in the presence of additives—DMC or DEC (Fig. 11 c, d)—represent peaks of grafted siloxane species at − 22, − 21, and − 19 ppm (Table 2) which are assigned to a mixture of D^2^ and D^1^ units in linear MD_4_M siloxane (Fig. 12b) and D^1^ unit in cyclotetrasiloxane (Fig. 12a), respectively. The grafting density for these surfaces is not as high as for surfaces modified with short (PMHS) and medium siloxane (PDMS) and representing values of 0.4 and 0.7 group/nm^2^ (Table 1), which are closer to “horizontal” chain orientation at the SiO_2_ surfaces. However, these values are three to five times higher than SiO_2_ modified with the neat polymer—CPDMS (*ρ*_max_ = 0.1 group/nm^2^, Table 1), and the data is in a good agreement with BET values (Table 1) which are lower for these samples than for SiO_2_/CPDMS one. A somewhat lower reactivity of neat CPDMS in relation to SiO_2_ surface could be attributed to the steric hindrance which could be caused by the long polymer chain units and epoxide groups which are present in this polymer, as long-chain organosiloxanes could form a helix structure [46, 47] due to the corresponding rotations around the Si–O bonds, which greatly limits the number of organosiloxane segments which are capable of interacting with active silica sites. On the other hand, in the concentrated solutions of organosiloxane in hexane, for example, the fraction of unfolded molecules increases [46], and this resulted in an increase in the density of contacts between the siloxane molecules and the SiO_2_ surface OH groups. Taking this into account, the use of an alkyl carbonate is beneficial, as under its influence the organosiloxane might change its structure, which in turn promotes the better accessibility of the formed polymer fragments to the silica surface silanols, and this promotes higher polymer adsorption at the SiO_2_ surface. The peaks broadening for SiO_2_/CPDMS+DMC (Fig. 11 c) and SiO_2_/CPDMS+DEC (Fig. 11 d) are due to a different steric orientation of the closely adjacent methyl and epoxy groups [34].

**Fig. 13:**
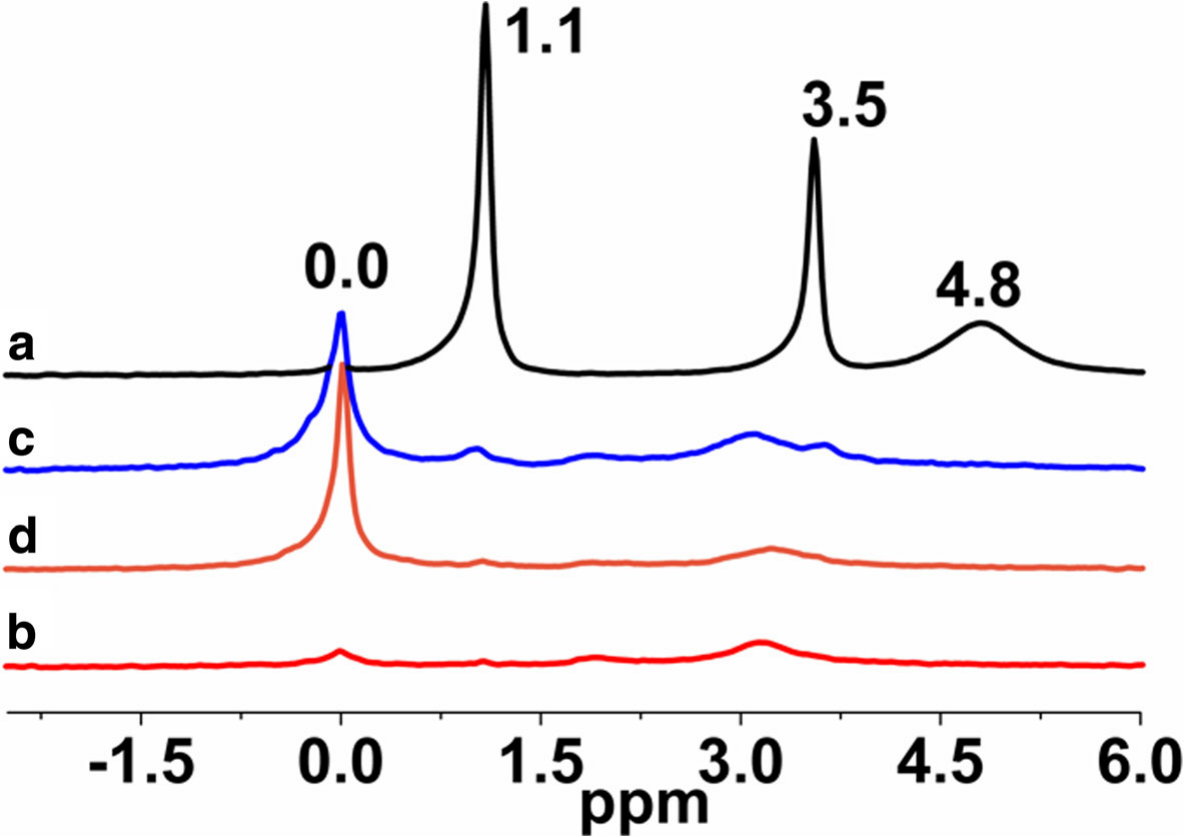
^1^H MAS NMR spectra of (**a**) neat fumed silica, (**b**) modified fumed silica with neat CPDMS, modified with (**c**) mixtures of CPDMS and dimethyl carbonate and (**d**) mixtures of CPDMS and diethyl carbonate

**Fig. 14:**
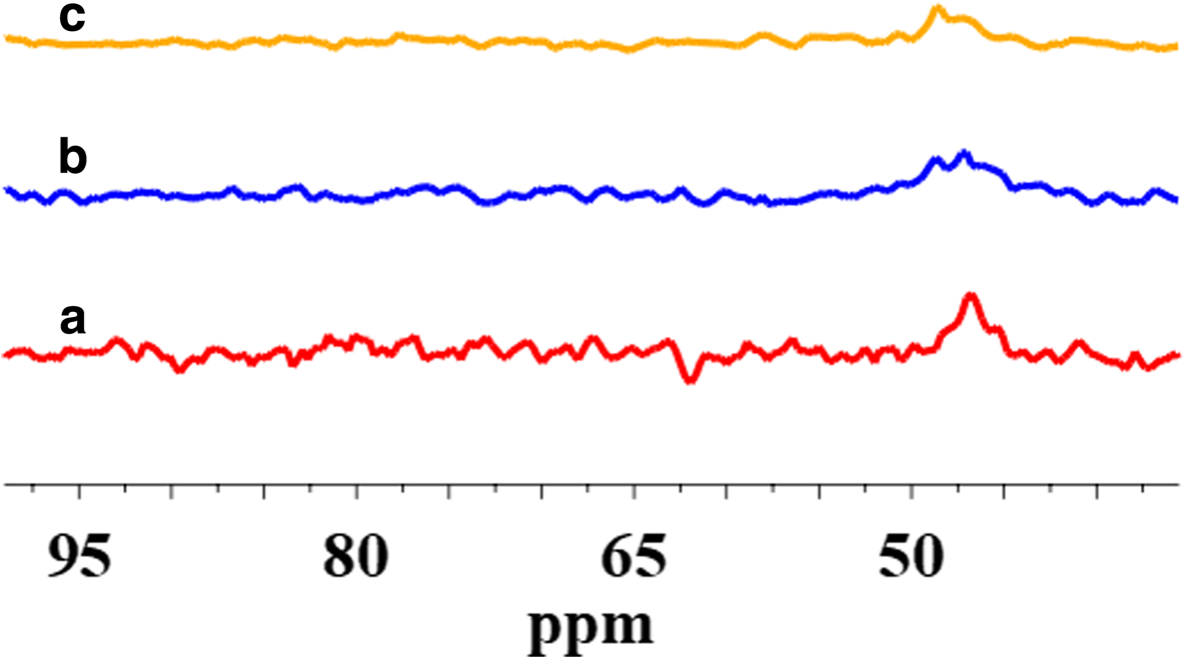
^13^C CP/MAS NMR spectra of (**a**) modified fumed silica with neat CPDMS, modified with (**b**) mixtures of CPDMS and dimethyl carbonate and (**c**) mixtures of CPDMS and diethyl carbonate

According to ^1^H MAS NMR, the spectra of the silicas which were modified by methyl-epoxy siloxane in the presence of DMC (Fig. 13 c) or DEC (Fig. 13 d) are nearly identical and in excellent agreement with ^29^Si CP/MAS NMR (Fig. 11 c, d). Grafted methyl-epoxy siloxane on silica surfaces for both samples resulted in shifts of 0.0 ppm and 3.2 ppm. The chemical shift at 3.2 ppm confirms the presence of the characteristic methy/epoxy groups for all the samples. In contrast, the resonance at 0.0 ppm for SiO_2_, modified with neat CPDMS (Fig. 13 b) is hardly detectable, which in accordance with the grafting density data (Table 1) demonstrates the small amount of long CPDMS units grafted at the SiO_2_ surface. Additionally, the ^13^C CP/MAS NMR data (Fig. 14) support this conclusion because only a very faint peak due to the alkyl groups at 44–50 ppm can be observed for the resulting samples. Note that this signal in the ^13^C CP/MAS NMR spectra of the presenting samples (Fig. 14) is broad, indicating a restricted mobility of the functional groups attached to the siloxane framework as discussed above.

Note that all the presented surfaces generally exhibit the grafting density decrease as the size of the polymer increases, used for surfaces functionalization. Similar results were also presented for silicas functionalized by different bis-fluoroalkyl disiloxanes [12]. This can be due to steric hindrance from the long polymer chains on the macromolecule, as discussed above.

## Conclusion

An in-depth solid-state NMR study of FSNs functionalized with organosiloxanes of various lengths of polymer chains and their mixtures of DMC or DEC is presented. For better analysis of the length of polymer chain effects, the organosiloxanes studied here are much longer and with a larger difference in the viscosity as well as pendant groups than the organosiloxanes studied before [12, 35, 48–54]. The obtained results reveal that the structure of the grafted species, type of grafting, and grafting density at the SiO_2_ surface depend strongly on the length of organosiloxane polymer and on the nature of the “green” additive, DMC or DEC. Spectral changes observed by solid-state NMR spectroscopy suggest that the major products of the reaction of various organosiloxanes and their DMC or DEC mixtures with the FSNs were D (RR’Si(O_0.5_)_2_) and T (RSi(O_0.5_)_3_) organosiloxane units. The appearance of grafted siloxane units at SiO_2_/PHMS+DEC and SiO_2_/PDMS+DMC surfaces is accompanied by a significant reduction of Q^3^ signals, while for neat organosiloxanes and some of their mixture with alkyl carbonate used for SiO_2_ modification, a reduction of Q^3^ is hardly observable. The small amounts of residual silanols (hardly accessible for modifier reagents used) and physisorbed water remain in all the samples of modified silicas (note that the crude silica was not preheated at high temperatures).

Addition of DMC to the modifying mixture facilitates the passage of chemical reaction between medium (PDMS) or long (CPDMS) polymer and the SiO_2_ surface. Diethyl carbonate addition somewhat worsens the chemical reaction between medium organosiloxane (PDMS) and SiO_2_ surface but greatly facilitates the reaction when organosiloxanes at short (PMHS) and long polymer chain (CPDMS) are applied for FSNs modification. Thus, from the technological point of view, for FSNs modification with short organosiloxanes, it is reasonable to use DEC; at medium organosiloxane, the application of DMC is necessary; and at long organosiloxane, it is beneficial to use both DMC and DEC.

The data for CP/MAS NMR, BET, and chemical analysis suggest the “vertical” orientation of grafted organosiloxane chains when short and medium polymer or its mixture with DMC (*ρ* = 7.2–7.4 groups/nm^2^) are applied for FSNs modification. The reaction of FSNs with medium and long polymer and its mixture with DEC (PDMS/DEC or CPDMS/DEC) leads to the formation of the “horizontal” chains at the surface (*ρ* = 0.1–2.5 groups/nm^2^). The findings open new ways for the preparation of similar materials of the same quality using different substrates such as various silicas—silica gels, porous silicas, and precipitated silica. The comparison of the influence of substrate nature on poly(organosiloxane)/alkyl carbonate modification is of undoubted interest for future study.

## Additional file

Additional file 1: **Figure S1.** 90 MHz ^1^H NMR spectrum of neat PMHS. **Figure S2.** 90 MHz ^1^H NMR spectrum of neat PDMS; the inset shows the methyl group shifts of parent PDMS. **Figure S3.** 90 MHz ^1^H NMR spectrum of neat CPDMS; the inset shows the methyl group shifts of parent CPDMS. (DOCX 1498 kb)

## Abbreviations

%*C*: Carbon weight percentage
CP/MAS NMR: Cross-polarization magic-angle spinning nuclear magnetic resonance
CPDMS: Poly[dimethylsiloxane-*co*-(2-(3,4-epoxycyclohexyl)ethyl)methylsiloxane]
DEC: Diethyl carbonate
DMC: Dimethyl carbonate
FSNs: Fumed silica nanoparticles
PDMS: Poly(dimethysiloxane)
PMHS: Poly(methylhydrosiloxane)
*S*_BET_: Surface area
SiO_2_: Silica
*δ*: Chemical shift
*ρ*: Bonding density
