# Supplementary material for: A 29Si, 1H, and 13C Solid-State NMR Study on the Surface Species of Various Depolymerized Organosiloxanes at Silica Surface
Source: Nanoscale Res Lett. 2019 May 14;14:160. doi: 10.1186/s11671-019-2982-2 (PMC6517472; doi:10.1186/s11671-019-2982-2)
Supplement: Supplementary file 1 — Figure S1. 90 MHz 1H NMR spectrum of neat PMHS. Figure S2. 90 MHz 1H NMR spectrum of neat PDMS; the inset shows the methyl group shifts of parent PDMS. Figure S3. 90 MHz 1H NMR spectrum of neat CPDMS; the inset shows the methyl group shifts of parent CPDMS. (DOCX 1498 kb) [file 11671_2019_2982_MOESM1_ESM.docx]

Additional file 1

| 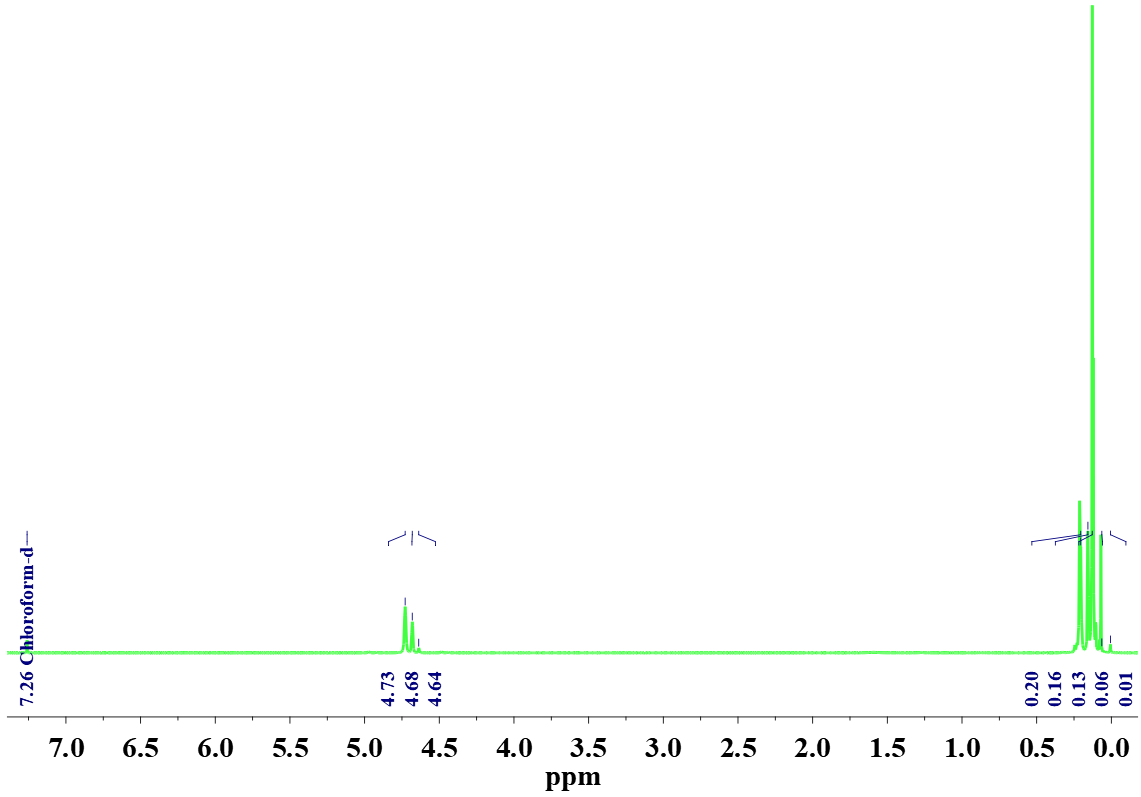 |
| --- |
| **Figure S1.** 90 MHz ^1^H NMR spectrum of neat PMHS. |

| ***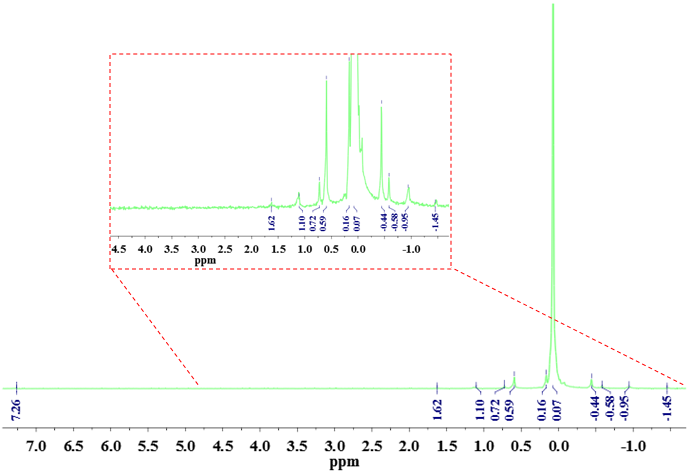*** |
| --- |
| **Figure S2.** 90 MHz ^1^H NMR spectrum of neat PDMS; the inset shows the methyl group shifts of parent PDMS. |

| 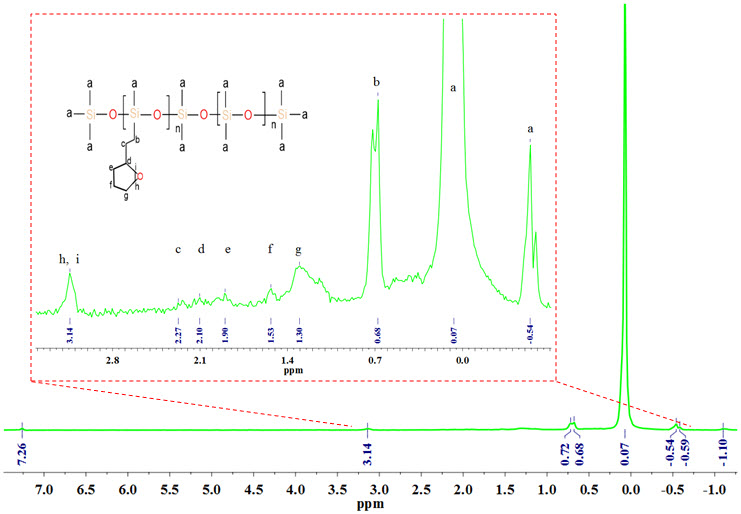 |
| --- |
| **Figure S3.** 90 MHz ^1^H NMR spectrum of neat CPDMS; the inset shows the methyl group shifts of parent CPDMS. |
